# The road from standardized to personalized medicine: the difficult-to-treat framework as a critical waystation

**DOI:** 10.3389/fmed.2026.1918272

**Published:** 2026-07-30

**Authors:** Lilla Gunkl-Tóth, Csaba Fűr-Kovács, György Nagy

**Affiliations:** 1 Department of Rheumatology and Immunology, Semmelweis University, Budapest, Hungary; 2 Department of Pharmacology and Pharmacotherapy, University of Pécs, Pécs, Hungary; 3 HUN-REN Chronic Pain Research Group, Pécs, Hungary; 4 AcademyEX Education Limited Partnership, Auckland, New Zealand; 5 Heart and Vascular Center, Semmelweis University, Budapest, Hungary; 6 Department of Genetics, Cell- and Immunobiology, Semmelweis University, Budapest, Hungary

**Keywords:** artificial intelligence, difficult-to-treat area, medicine, personalized medicine, precision medicine

## Abstract

Standardized care has become the cornerstone of modern medicine. However, many patients remain symptomatic despite following the appropriate guidelines and the application of treatment escalation, and become difficult-to-treat (D2T). The D2T concept can also provide a framework for identifying when standard care is no longer sufficient and when individualized management becomes clinically justified. Rather than reflecting therapeutic failure alone, the D2T state can be a consequence of various reasons, including underlying biological, clinical, psychosocial, or contextual complexity, which draws attention to the importance of multidomain reassessment in these conditions. Combined with emerging artificial intelligence-based trajectory recognition, the D2T framework may support earlier identification of complex patients and more selective implementation of precision strategies. In this way, the D2T concept can be understood as a practical bridge between standardized medicine and individualized care, with a potential relevance as a cross-disciplinary framework.

## Introduction

Contemporary medicine is built on a productive but still unresolved tension between standardization and individualization. Over recent decades, evidence-based medicine (EBM) has transformed clinical care by deriving treatment strategies from large-scale clinical trials representing population-level evidence and embedding them in structured management frameworks and generalizable guidelines. In chronic conditions, this logic has often been implemented through treat-to-target approaches that define clear, measurable therapeutic goals, and guide treatment escalation. However, these advances have also exposed a persistent subset of patients who remain symptomatic despite following every guideline-based recommendation. The emergence of the difficult-to-treat (D2T) construct reflects this limitation of standardized medicine and suggests that, in some patients, failure of the average pathway should prompt a transition to a more individualized model of care (1)

## When standard is not enough

**Figure 1:**
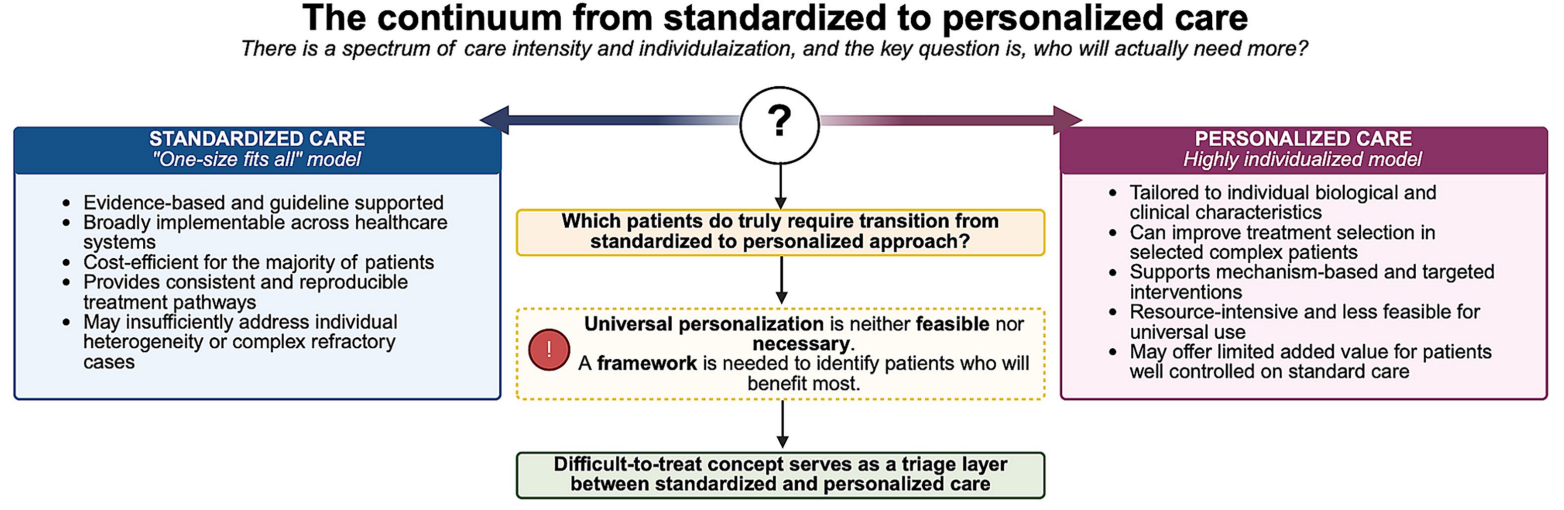
The D2T concept serves as a triage framework between standardized and personalized care. Standardized, guideline-based care is effective and scalable for the majority of patients but may fail to capture individual heterogeneity in more complex cases. While personalized care offers tailored, mechanism-based interventions, it is resource-intensive and not required for all patients. The central clinical challenge is to identify which patients truly require transition along this spectrum. As universal personalization is neither feasible nor necessary, the D2T concept provides a structured framework to recognize patients in whom standard care is insufficient, thereby guiding selective and timely implementation of individualized management strategies.

Standardized treatment algorithms form the basis of EBM, and their use leads to adequate disease control across many different medical conditions. For instance, first-line antihypertensive medications such as diuretics or angiotensin-converting enzyme (ACE) inhibitors are widely used and have proven effective for most patients, whereas standard inhaled corticosteroid-based regimens provide satisfactory disease control for many individuals with asthma (1–3). In addition to their effectiveness, these approaches are scalable and relatively inexpensive, which helps explain why they can be key components of guideline-based care. This means that comprehensive and resource-intensive methods, including individual genomic or molecular profiling to choose a more individualized strategy, are not always necessary in clinical decision-making, underscoring that, despite its future potential, personalized medicine may not be the appropriate initial approach for all patients (Figure 1).

However, the limitations of standardized treatment algorithms become apparent in patients who remain symptomatic despite sequential, guideline-based treatment and develop a therapy-resistant condition, that is D2T. At the same time, symptomatic improvement does not necessarily indicate resolution of the underlying pathophysiological processes, which may persist despite apparent clinical improvement and, if left unrecognized or inadequately treated, may lead to irreversible consequences. Furthermore, it is also important to acknowledge that the clinical problem often extends beyond insufficient response to a given drug, but symptom persistence may instead reflect the interaction of several factors, including biological heterogeneity, comorbidities, psychosocial context, and other forms of patient-level complexity that fall outside the assumptions of standardized guidelines (1). This broader view also highlights the need to look beyond symptoms alone. Where available and appropriately validated, biomarkers can also add important information to this multidimensional assessment and help to better characterize the mechanisms underlying the D2T state.

**Figure 2:**
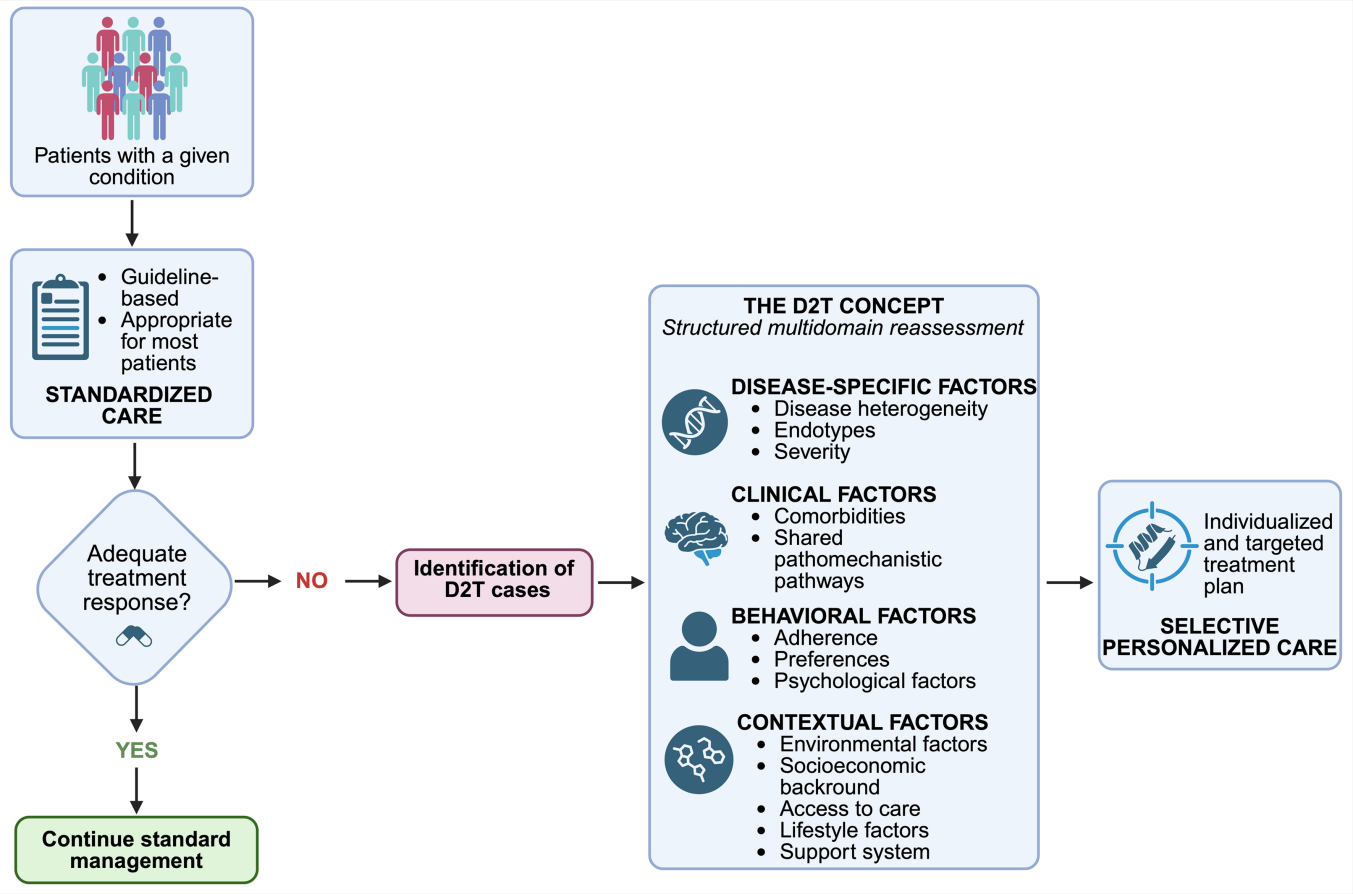
The figure introduces the operational pathway of the D2T concept in clinical practice. Patients initially receive guideline-based standardized care, which is sufficient for most individuals. In cases of inadequate treatment response, patients are identified as potential D2T cases and should undergo structured, multidomain reassessment. This process integrates disease-specific, clinical, behavioral and contextual factors to better understand the drivers of non-response. The outcome of this evaluation is not universal escalation, but the development of a targeted, individualized management strategy for selected patients.

However, the mechanisms contributing to the D2T state should not be considered in isolation. If we consider these factors as a network, we can conceptualize disease phenotypes not as the consequence of a single isolated abnormality, but as the result of perturbations within interconnected biological systems (4). In the D2T context, these interacting determinants should therefore be identified and addressed not only individually but also in relation to one another, as changes in one component could induce broader, network-level consequences shaping disease trajectory; thus, necessitating a more complex view and assessment. The D2T concept, therefore, can represent a shift in perspective, as it should not be considered simply a marker of therapeutic refractoriness, but rather an approach that identifies patients in whom standard, guideline-based care is no longer efficient and a more individualized strategy may be required (5). In this context, the D2T approach may serve as an operational linkage between general, one-size-fits-all guidelines and personalized medicine, helping to define the subgroup for whom more detailed phenotyping and tailored management are needed (Figure 2).

## D2T across the medical landscape

Across medicine, clinicians recognize a common subgroup of patients whose disease remains persistently difficult to control despite guideline-based care, and many definitions have been developed to describe this population, ranging from terms describing treatment refractoriness and difficulties in management (1). The clinical importance of identifying such patients is especially evident in chronic diseases, where prolonged inadequate disease control may lead to cumulative morbidity, irreversible damage and premature mortality.

In asthma, the distinction is more hierarchical: D2T asthma denotes the broader state of persistent poor control that prompts reassessment, whereas severe asthma refers to the subset that remains uncontrolled despite optimized treatment and correction of contributory factors (2). In hypertension, the analogous state is resistant hypertension, where failure to achieve targets redirects attention from routine escalation to secondary or remediable causes (3). In psychiatry, for example, in the case of depression, treatment resistance is interpreted less as a single biological state than as evidence of heterogeneity in pathophysiology, comorbidity, trauma exposure or drug metabolism (6). In chronic or recurrent infection, the D2T disease is often framed around multidrug-resistant pathogens, but therapeutic failure may also arise from biofilm-associated tolerance and host susceptibility; persistent infection therefore requires reassessment of both microbial and host factors, not only drug resistance (7, 8). In oncology, the same problem is operationalized through molecular stratification, where standard treatment pathways give way to biomarker-guided therapy once they are no longer sufficiently informative (9). In rheumatology, this has been formalized most explicitly in D2T rheumatoid arthritis, where persistent disease despite multiple therapies requires reassessment of both inflammatory and non-inflammatory contributors (10). Taken together, these examples suggest that the D2T concept is best understood not as a single disease model, but as a cross-disciplinary framework whose meaning is defined locally by how each specialty explains, verifies and manages failure of the standard pathway.

## A cost-effective model of precision

The connection between the D2T concept and personalized medicine is best understood through the logic of stratified efficiency. Precision medicine is unlikely to be clinically sustainable if deployed indiscriminately across entire populations; rather, its value depends on identifying who would benefit most from a more complex assessment and intervention. Recent frameworks for evidence-based precision medicine make this point explicitly, arguing that precision approaches must be benchmarked against contemporary care and judged not only by innovation, but by clinical utility, scalability and societal benefit (11). In this context, the D2T framework can be viewed as a gatekeeping layer for personalized medicine: a way of identifying patients whose trajectory is no longer adequately captured by standard care, and who therefore justify deeper phenotyping or more individualized treatment. This is also economically rational. Precision trial designs increasingly use staged enrichment strategies to focus costly profiling on those most likely to benefit, improving efficiency and resource allocation, while avoiding the impracticality and inequity of applying expensive omics-based approaches to everyone (11).

## The role of AI in identifying complexity

The central challenge of the D2T concept is not simply to describe complexity once it has fully emerged, but it rests on our ability to predict who will become D2T before they endure years of failed therapies. In that sense, D2T is fundamentally a problem of trajectory recognition: identifying which patients are beginning to diverge from the path that standardized, guideline-based care would normally predict. This is precisely where modern technologies, especially artificial intelligence (AI), become clinically relevant. AI and machine-learning algorithms are uniquely capable of synthesizing “big data” from electronic health records, wearable devices, and multi-omic data registries. Rather than operating autonomously, AI adds the most clinical value within a doctor-patient-AI triad, integrating complex data streams to assist in highly patient-specific decisions (12). Importantly, domain-specific AI systems trained on clinical data have already shown the ability to forecast future medical events and diagnoses from electronic health records, indicating that AI can move beyond description toward prediction (13). Applied to the D2T framework, this would mean using AI not to personalize therapy for everyone from the outset, but to detect, earlier and more reproducibly, the subgroup of patients whose disease trajectory is already departing from the standard pathway and who may therefore require earlier escalation or a more individualized therapeutic strategy (12).

Recent clinical studies from different medical fields validate the successful implementation of AI technologies into existing workflows where standard care underperforms. In breast cancer screening, the PRAIM study showed that integrating AI into routine mammography reading increased cancer detection by 17.6% without increasing recall rates, illustrating that AI can improve performance when embedded into an existing clinical workflow rather than used as a stand-alone replacement (14). Similarly, in the TAILORED-AF randomized controlled trial, investigators addressed another relevant clinical problem: while pulmonary vein isolation is the standard ablation strategy for atrial fibrillation, outcomes in persistent and long-standing persistent disease remain suboptimal. The trial, therefore, tested whether AI could identify spatio-temporal electrogram dispersion patterns that would allow a more individualized intervention. In 374 randomized patients, the AI-guided tailored strategy achieved the primary endpoint in 88% of patients compared with 70% in the conventional arm, with no difference in the composite safety endpoint, although the procedures were longer. The importance of this study is not only that AI improved performance, but also how it did so: it helped move beyond a uniform strategy in a clinical setting where the standard approach is known to underperform (15). A similar principle appears in a randomized study of AMIE in complex cardiology care, also demonstrating a predictive edge. There, nine general cardiologists assessed 107 real-world cases of suspected genetic cardiomyopathy with or without large language models (LLM) assistance using multimodal data, including ECG, echocardiography, cardiac MRI and exercise testing. Blinded subspecialists preferred the AI-assisted assessments overall more often than unassisted ones (46.7% versus 32.7%), and the AI-assisted arm had fewer clinically significant errors and fewer omissions of important information (16). Although these studies illustrate the potential of AI in selected clinical settings, their findings cannot yet be directly generalized, as prospective validation and evidence of clinical utility are needed. Nevertheless, these results point to a recurring clinical scenario in which AI may be especially valuable: the identification of complex cases reaching the limits of standard care. This capacity to detect clinically meaningful divergence from expected pathways provides the rationale for extending AI-supported prediction to the D2T domain.

Transitioning this predictive capability to the D2T domain requires a sophisticated level of multimodal data integration that conventional clinical evaluation cannot easily achieve alone, precisely because D2T trajectories can be understood as a network of interacting biological, clinical, behavioral, and healthcare-system determinants rather than by isolated variables (1, 4). Electronic health records contain longitudinal information on diagnoses, treatments, examination results, and healthcare utilization, while wearable devices and digital tools may add information on sleep, physical activity, adherence, and other lifestyle-related factors. In more integrated healthcare systems, socioeconomic and behavioral data may also become available, which would further add potential advantage, as these factors can strongly influence treatment response, access to care, and long-term outcomes. For an individual clinician, synthesizing all this data over time is almost impossible. But for data-driven models, such information can be analyzed more easily and presented in an easy-to-review user interface, for example, as a heatmap, or other dynamic risk-trajectory visualization, to reduce cognitive burden on healthcare professionals (17). This visual synthesis and the underlying data add valuable details to the calculation of a broader trajectory. The value of AI in the D2T framework would therefore lie in detecting early patterns of complexity before they become clinically obvious. In a D2T framework, specialty-specific AI tools could support early detection of resistant hypertension, severe asthma, treatment-resistant depression, or inflammatory disease flares by combining temporal patterns, prior treatment response, and contextual data (18). In principle, this could shift D2T from a retrospective label applied after exhaustion of standardized algorithms to a prospective risk estimate used earlier in the patient journey. This would enable us to identify the subgroup in whom standard care is unlikely to be sufficient and benefit from a more personalized approach. In the future, at early stages of the triage, AI could be engaged to flag patients likely to require precision medicine, because increasingly sophisticated and cost-effective models may make such screening feasible at scale (19, 20). However, the transition from promising exploratory findings to clinical application requires rigorous validation in independent populations and evidence that these models provide meaningful clinical benefit. Furthermore, AI-aided support should remain clinician-supervised and integrated into explainable, human-in-the-loop workflows to avoid mistakes caused by AI, to secure clinician trust, and preserve accountability (21, 22).

## Conclusion

The D2T framework teaches us that uniform success requires individualized understanding. By using contemporary treatment strategies as a screening tool, healthcare systems can identify the more complex patients early. Integrating personalized medicine at this specific intervention point ensures that the most sophisticated, and often most expensive, tools of modern science are delivered to the right person at the right time, transforming the D2T concept from a label of treatment failure into a roadmap for recovery.

## Data Availability

The original contributions presented in the study are included in the article/supplementary material, further inquiries can be directed to the corresponding author.
